# Expected and unexpected evolution of plant RNA editing factors CLB19, CRR28 and RARE1: retention of CLB19 despite a phylogenetically deep loss of its two known editing targets in Poaceae

**DOI:** 10.1186/s12862-018-1203-4

**Published:** 2018-06-07

**Authors:** Anke Hein, Volker Knoop

**Affiliations:** IZMB – Institut für Zelluläre und Molekulare Botanik, Abteilung Molekulare Evolution, Universität Bonn, Kirschallee 1, D-53115 Bonn, Germany

**Keywords:** RNA-binding PPR proteins, Plant mitochondria and chloroplasts, Angiosperm evolution

## Abstract

**Background:**

C-to-U RNA editing in mitochondria and chloroplasts and the nuclear-encoded, RNA-binding PPR proteins acting as editing factors present a wide field of co-evolution between the different genetic systems in a plant cell. Recent studies on chloroplast editing factors RARE1 and CRR28 addressing one or two chloroplast editing sites, respectively, found them strictly conserved among 65 flowering plants as long as one of their RNA editing targets remained present.

**Results:**

Extending the earlier sampling to 117 angiosperms with high-quality genome or transcriptome data, we find more evidence confirming previous conclusions but now also identify cases for expected evolutionary transition states such as retention of RARE1 despite loss of its editing target or the degeneration of CRR28 truncating its carboxyterminal DYW domain. The extended angiosperm set was now used to explore CLB19, an “E+”-type PPR editing factor targeting two chloroplast editing sites, rpoAeU200SF and clpPeU559HY, in *Arabidopsis thaliana*. We found CLB19 consistently conserved if one of the two targets was retained and three independent losses of CLB19 after elimination of both targets. The Ericales show independent regains of the ancestrally lost clpPeU559HY editing, further explaining why multiple-target editing factors are lost much more rarely than single target factors like RARE1. The retention of CLB19 despite loss of both editing targets in some Ericaceae, Apocynaceae and in *Camptotheca* (Nyssaceae) likely represents evolutionary transitions. However, the retention of CLB19 after a phylogenetic deep loss in the Poaceae rather suggests a yet unrecognized further editing target, for which we suggest editing event ndhAeU473SL.

**Conclusion:**

Extending the scope of studies on plant organelle RNA editing to further taxa and additional nuclear cofactors reveals expected evolutionary transitions, strikingly different evolutionary dynamics for multiple-target editing factors like CLB19 and CRR28 and suggests additional functions for editing factor CLB19 among the Poaceae.

**Electronic supplementary material:**

The online version of this article (10.1186/s12862-018-1203-4) contains supplementary material, which is available to authorized users.

## Background

The simultaneous existence of three separate genomes in the chloroplast, mitochondrion and nucleus in every plant cell requires co-adaptation and co-evolution for successful and co-ordinated gene expression [1]. Genetic incompatibilities between the nuclear genetic system and those in the two endosymbiotic organelles result in malfunctions during a plant’s lifecycle. A prime example for such incompatibilities among flowering plants is the phenomenon of cytoplasmic male sterility (CMS), a trait of significant interest to produce hybrid seeds in plant breeding and agronomy. In CMS lines, the lack of appropriate nuclear restorer genes fails to suppress deleterious gene products in the mitochondria leading to their malfunction during pollen biogenesis. Male-fertile plants require crossing with appropriate restorer lines to adequately control mitochondrial gene expression [2].

A particularly wide field of investigation for nucleus-organelle co-ordination and co-evolution has emerged with the identification of specific RNA editing factors addressing the numerous sites of C-to-U RNA editing in plant chloroplasts and mitochondria. Essentially, the individual sites of RNA editing in the two organelle transcriptomes are targeted by a special class of RNA-binding pentatricopeptide repeat (PPR) proteins [3, 4]. These “PLS-type” PPR proteins serving as editing factors are composed of an organelle targeting signal, an array of tandemly arranged P-, L- and S-type PPRs specifically recognizing an RNA sequence target and three carboxyterminal protein domains, recently re-defined and re-designated as E1, E2 and DYW [5]. After the initial characterization of CRR4 as a first chloroplast [6] and MEF1 as a first mitochondrial editing factor [7] in the model angiosperm *Arabidopsis thaliana*, more than 70 PLS-type editing factors addressing individual or multiple sites in chloroplasts or mitochondria have been identified.

The ultimate carboxyterminal DYW domain in most editing factors has received particular attention owing to its evident similarity with cytidine deaminases [8–11] and its strict co-existence with (mitochondrial) RNA editing within and outside of the plant kingdom [12, 13]. However, whereas all editing factors in the model moss *Physcomitrella patens* carry the full suite of carboxyterminal domains E1-E2-DYW [14–16], many of the site-specific recognition factors in flowering plants appear truncated, lacking a complete DYW domain.

The growing list of flowering plants for which high quality genome (and/or transcriptome) data have become available should allow to trace the co-evolution of editing factors and their cognate RNA editing targets. Of interest in that regard are ancient RNA editing factors emerging early in the evolutionary history of flowering plants that allow to track their evolutionary fate and those of their cognate editing sites for ca. 140 million years of flowering plant diversification. We recently found that in contrast to only moderate chloroplast RNA editing in typically investigated angiosperm models like *Arabidopsis thaliana, Nicotiana tabacum* or *Oryza sativa* with some 30–50 editing sites, editing is much more abundant in the chloroplast transcriptomes of early-branching angiosperms such as *Amborella trichopoda* with more than 130 chloroplast edits [17]. Interestingly, most chloroplast RNA editing sites in *Arabidopsis* (20 of 32) were also identified in *Amborella*, possibly indicating their very ancient origin among angiosperms. Moreover, those shared chloroplast editing sites included some, for which a specific editing factor had already been identified in *Arabidopsis*, including CRR28 and RARE1, which opened the possibility to identify editing factor orthologues and trace their evolutionary history. Defining a 65 taxon-set of angiosperms with high-quality genome (and/or transcriptome) data available, we indeed consistently identified CRR28 and RARE1 orthologues in all angiosperms where a requirement for editing remained at the respective chloroplast targets. Intriguingly, RARE1 concomitantly disappeared with loss of its editing target accDeU794SL through C-to-T conversion at DNA level at least 14 times independently [17]. In contrast, CRR28 was retained in all six observed cases of losing either the one or the other of its two target sites (ndhBeU467PL and ndhDeU878SL) and was found uniquely lacking only in chickpea (*Cicer arietinum*) once both original editing targets were simultaneously converted into thymidines in the cpDNA making C-to-U editing at these two sites obsolete. These observations suggested that the nuclear-encoded RNA editing factors in plants may disappear surprisingly quickly once the necessity for editing at their organelle target sites is lost and that a single-target editing factor like RARE1 gets lost more frequently than a multiple-target RNA editing factor like CRR28.

To further evaluate those conclusions, we investigated additional high-quality angiosperm genome and transcriptome data that have become available, thus significantly extending the original 65 taxa sampling into a 117 angiosperm species data set. The widely extended taxon sampling largely corroborates the above conclusions. We identified four new cases for simultaneous loss of editing site accDeU794SL and its corresponding editing factor RARE1. In contrast, we found that CRR28 was consistently retained in seven new cases of losing either the one or the other of its two editing targets ndhBeU467PL or ndhDeU878SL.

Given the intriguing results on CRR28 we now additionally investigated CLB19 as another chloroplast editing factor previously shown to address two chloroplast editing sites simultaneously in *Arabidopsis thaliana*: clpPeU559HY and rpoAeU200SF [18]. We identified three cases where both CLB19 editing target sites are converted into thymidines making editing obsolete and these cases perfectly coincide with an apparent absence of CLB19 orthologues in the nuclear genomes, hence analogous to the previously identified case of CRR28 in chickpea. Inspecting the evolution of CLB19 more closely among the Ericales reveals an intriguing loss and re-gain scenario for the cognate editing sites. Moreover, and in stark contrast to CRR28, we now also observe cases where CLB19 orthologues are retained despite the loss of both known editing target sites, most notably in all Poaceae. We assume that evolutionary pressure for an additional, yet unrecognized function of CLB19 is key to explain its retention for millions of years in that case.

## Results

### Extending the angiosperm taxon sampling

**Fig. 1:**
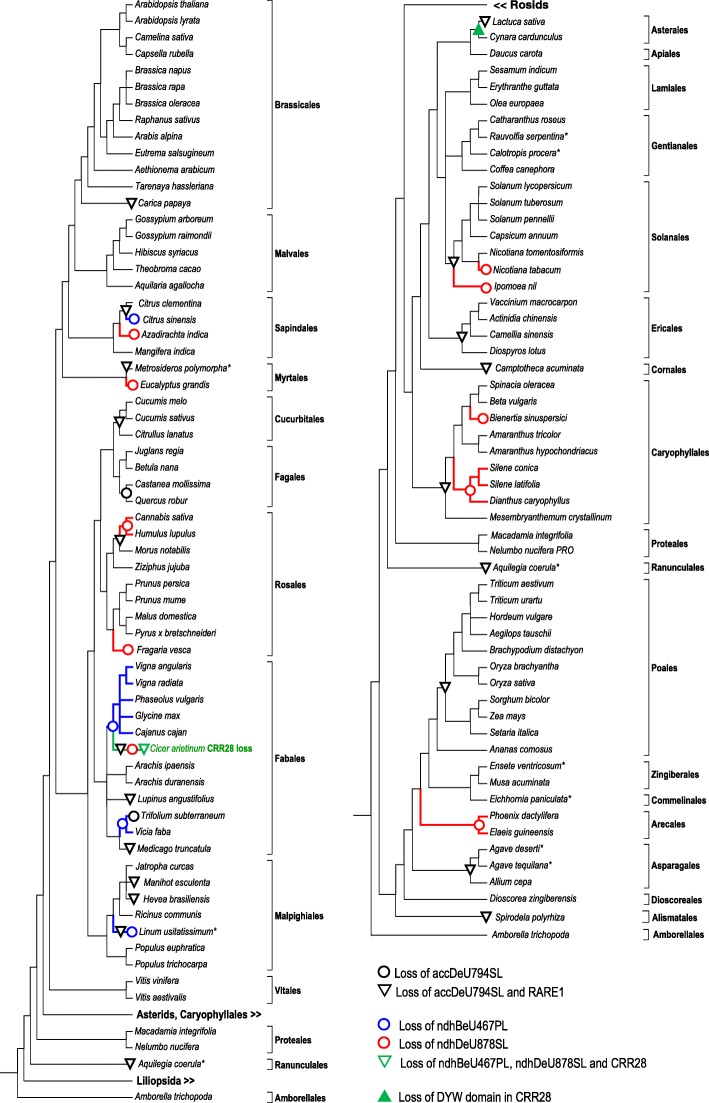
Cladograms for a sampling of 117 angiosperm species according to the current understanding of flowering plant phylogeny. The large clades of Asterids, Caryophyllales and Liliopsida (monocots) are collapsed in the left cladogram and, vice versa*,* the large clade of Rosids is collapsed in the right cladogram. Species marked with asterisks lack complete chloroplast genome data. Closely related cpDNAs were inspected in these cases or chloroplast targets have been individually investigated in this study (here e.g. *ndhB* and *ndhD* sequences of *Rauvolfia* and *Diospyros*). Black downward triangles indicate simultaneous losses of editing site accDeU794SL and editing factor RARE1. The black circles indicate loss of the accDeU794SL editing site in *Quercus* and *Castanea* (Fagaceae, Fagales) or of the *accD* gene altogether (*Trifolium*) while RARE1 orthologues remain present. Blue circles indicate independent losses of editing site ndhBeU467PL owing to a plastomic C-to-T conversion in *Linum*, *Vicia*, *Trifolium*, a Fabales subclade including *Cicer,* and in *Citrus sinensis*. Red circles indicate loss of the ndhDeU878SL editing site in Arecales (*Phoenix* and *Elaeis*), in *Dianthus*, *Silene* and *Bienertia* among Caryophyllales, in *Ipomoea* and *Nicotiana tabacum* (Solanales), in *Cicer*, *Fragaria*, *Cannabis* and *Humulus*, in *Eucalyptus* and in *Azadirachta*. CRR28 orthologues were identified in all taxa except in chickpea (*Cicer arietinum*) were both editing sites are lost (green downward triangle). The green upward triangle indicates truncation of CRR28 behind the E2 domain in *Cynara* and *Lactuca*. Phylograms of CRR28 and RARE1 orthologues are available as Additional files 1 and 2, respectively

We re-applied our previous criteria originally resulting in a 65-angiosperm data set [17] to identify additional flowering plant species with high-quality sequence data that have become available. Further species were included in our extended taxon sampling either when obviously reliable protein models were available, when somewhat less reliable protein models could be amended with corrected translations from whole genome shotgun or transcript shotgun assembly data or when entirely new de novo translations could be deduced clearly after identifying TBLASTN hits in the WGS or TSA databases at the NCBI (www.ncbi.nlm.nih.gov). Ultimately, we thus expanded our sampling to now comprise 117 angiosperms included in the cladograms discussed in the following (Figs. 1 and 3). The new taxon set now includes species from six angiosperm orders that were previously not represented (Apiales, Asterales, Cornales, Commelinales, Dioscoreales and Ranunculales), altogether now including 28 of 64 recognized angiosperm orders. Several of the new species are of special interest given their crucial phylogenetic positions. Examples are early-diverging monocots like *Dioscorea zingiberensis* or the pineapple *Ananas comosus* as a sister taxon to the Poaceae. *Aquilegia coerula* and *Macadamia integrifolia* are added as further representatives of early diverging eudicots. The campanulid clade within the asterids is now represented by carrot (*Daucus carota)*, cardoon (*Cynara cardunculus)* and lettuce (*Lactuca sativa).* Other important addendums are the elevated number of Caryophyllales species, especially of the *Silene* genus which is known to be highly variable in mitochondrial RNA editing [19, 20] and taxa representing additional families in large orders such as the Cannabaceae in the Rosales or the Anacardiaceae in the Sapindales. Finally, *Arachis hypogea* and *Gossypium hirsutum* were replaced with closely related species *A. duranensis, A. ipaensis, G. arboreum and G. raimondii*, respectively, which offered yet better sequence qualities.

### CRR28 and its editing targets ndhBeU467PL and ndhDeU878SL

Like in our previous study we consistently identified highly conserved CRR28 orthologues in all newly added taxa if at least one of its two known RNA editing target sites was present in the chloroplast DNAs (Fig. 1, Additional file 1). In addition to the previously detected losses of ndhDeU878SL in *Eucalyptus, Fragaria*, *Cicer*, *Nicotiana tabacum* and in the palms (Arecales) we now observed five additional independent losses of this editing site in *Azadirachta*, in *Cannabis* and *Humulus*, in *Dianthus* and *Silene*, in *Bienertia* and in *Ipomoea* (Fig. 1). In contrast, other than the two previously reported losses of ndhBeU467PL editing in *Linum* and in the *Cicer*/Phaseolae clade among Fabales, only two additional independent losses are now identified in *Citrus sinensis* and in *Vicia* and *Trifolium,* which at present remain phylogenetically unresolved among Fabales. Hence, the extended data set supports the previous finding that editing target site ndhDeU878SL is lost more frequently than ndhBeU467PL (10 vs. 4–5 independent losses). In all these additional cases of losing either the one or the other editing target site, CRR28 orthologues are retained, evidently because the respective other editing site needs to be addressed. The extended CRR28 protein phylogeny is in full agreement with the species phylogeny indicating common orthologue ancestry (Additional file 1). Intriguingly, *Cicer arietinum* (chickpea) remains the only case of a double loss of both RNA editing sites and the only case where no CRR28 homologue could be detected (Fig. 1).

CRR28 is a “DYW-type” RNA editing factor featuring the full set of carboxyterminal extra domains E1, E2, and DYW behind the PPR array for RNA sequence recognition in nearly all taxa. The CRR28 orthologues now identified in *Cynara* and *Lactuca* (Asterales), however, are truncated behind the so-called “PG-box” at the beginning of the DYW domain (Fig. 1). The truncated CRR28 proteins in the Asterales are likely functionally reduced to RNA target recognition and now require provision of a cytidine deaminase activity *in trans* (see discussion and Fig. 6).

### RARE1 and its editing target accDeU794SL

The extended flowering plant sampling now suggests the previously identified absence of the accDeU794SL editing target in *Beta, Actinidia* and *Vaccinium* as well as in *Nicotiana* and *Solanum* to represent ancient losses deep in the respective orders Caryophyllales, Ericales and Solanales, respectively (Fig. 1). Adding to the previously identified 15 independent losses of editing site accDeU794SL, new cases were identified in the Cannabaceae (*Humulus* and *Cannabis*), in *Lactuca,* in the Ranunculales (*Aquilegia*), in the Myrtales (*Metrosideros)* and in the Fagaceae (*Castanea* and *Quercus*). Note that instead of a C-to-T conversion in the chloroplast gene copy, the loss of editing may alternatively occur (e.g. in Poaceae or in *Trifolium subterraneum*) as a result of endosymbiotic gene transfer of *accD* to the nucleus [21, 22]. Given the sister group placement of Cannabaceae to the previously identified case of *Morus,* the extended sampling identified a total of ca. 20 independent losses of the accDeU794SL editing site (Fig. 1).

In the clear majority of the more than 50 angiosperms in our sampling which lack the accDeU794SL editing site, this is linked to the simultaneous absence of editing factor RARE1 in the genomic data. However, we now find that RARE1 is retained in the genomes of oak (*Quercus*) and chestnut (*Castanea*), the two Fagaceae species (Fagales) in the extended taxon sampling, although a C-to-T conversion in their cpDNAs makes RNA editing obsolete at the previous accDeU794SL editing site (Fig. 1, Additional file 2). Similarly, RARE1 is retained in *Trifolium subterraneum* although the functional *accD* gene is lost from the cpDNA. The RARE1 orthologues in the Fagaceae and in *Trifolium* show no signs of degeneration into pseudogenes (including the crucial PPR positions 5 and L (“Last”) for RNA target recognition, see below) arguing for a comparatively recent evolutionary loss of the editing target by C-to-T conversion or *accD* transfer to the nucleus. This is a likely scenario for the dynamic cpDNA evolution in the genus *Trifolium* [22], whereas a loss of the accDeU794SL editing target in the common ancestor of *Castanea* and *Quercus* ancestor would be dated to approximately 4 mio. Years ago ([23], see www.timetree.org).

### CLB19 and its editing targets clpPeU559HY and rpoAeU200SF

We wished to investigate whether the conservation of editing factor CRR28 targeting two chloroplast RNA editing sites simultaneously is an exceptional case in angiosperm evolution. CLB19 was characterized as another RNA editing factor also addressing two chloroplast RNA editing sites at the same time [18]. Its two corresponding target sites (clpPeU559HY and rpoAeU200SF) were likewise found to be conserved between *Arabidopsis thaliana* and *Amborella trichopoda* [17].

**Fig. 2:**
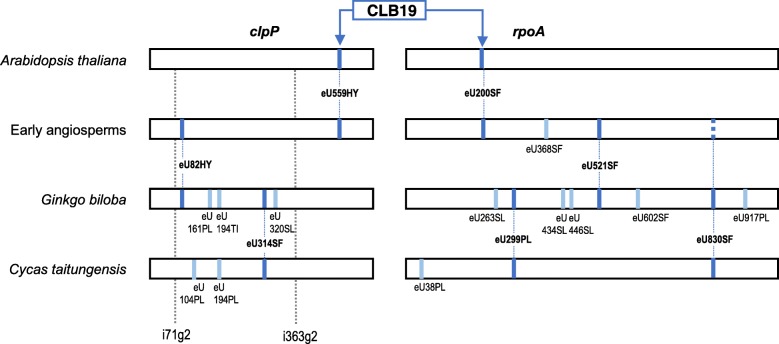
Overview on RNA editing in transcripts of the chloroplast genes *clpP* and *rpoA.* Editing sites clpPeU559HY and rpoAeU200SF are the only editing events in *Arabidopsis thaliana.* They are shared in early-diverging angiosperms like *Amborella trichopoda, Illicium oligandrum* and *Chloranthus spicatus*, which feature up to four additional sites of editing. Editing site rpoAeU830SF (dotted line) is present in *Illicium* and *Chloranthus*, but not in *Amborella*. Only editing sites clpPeU82HY, rpoAeU521SF and rpoAeU830SF have counterparts in the gymnosperms *Ginkgo biloba* and *Cycas taitungensis* suggesting that edits clpPeU559HY and rpoAeU200SF and their cognate editing factor CLB19 could be a molecular synapomorphy of angiosperms. Dotted grey lines indicate positions of the two conserved group II introns clpPi71g2 and clpPi363g2 in the land plant chloroplast genomes

**Fig. 3:**
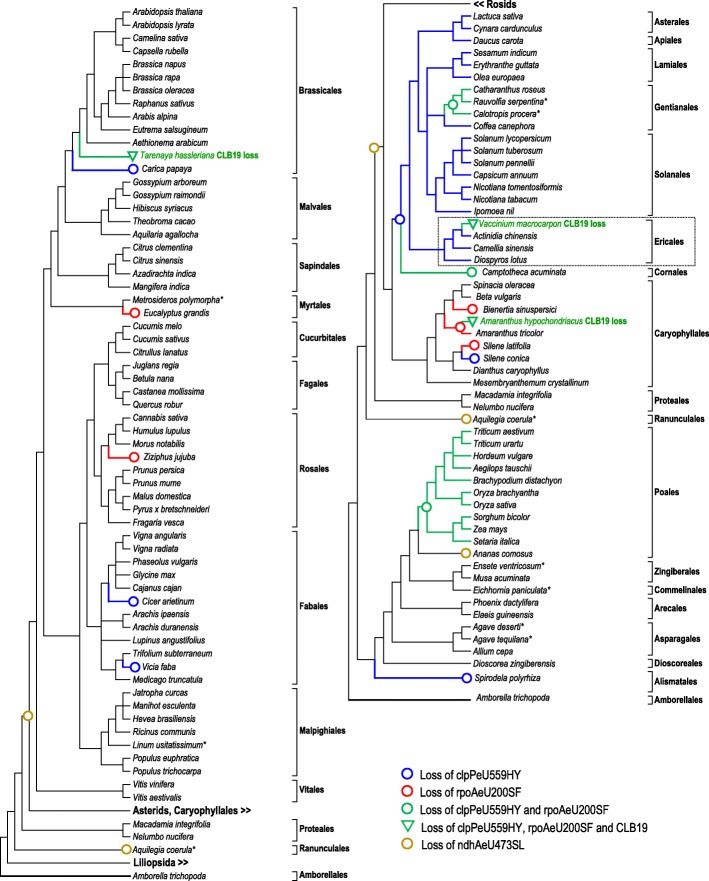
Cladograms of 117 angiosperms as shown in Fig. 1. Red open circles indicate loss of chloroplast RNA editing site rpoAeU200SF (in *Ziziphus, Eucalyptus, Amaranthus tricolor, Bienertia* and *Silene latifolia*). Blue open circles indicate loss of editing site clpPeU559HY (in *Vicia, Cicer, Carica*, *Silene conica, Spirodela* and in the Asterid clade). Both RNA editing sites simultaneously (green symbols) are absent in the Poales*,* in *Amaranthus hypochondriacus*, *Camptotheca acuminata, Vaccinium macrocarpon* in the Apocynaceae (*Calotropis, Catharantus, Rauvolfia*) and in *Tarenaya hassleriana*. No orthologues of editing factor CLB19 could be identified in *Amaranthus hypochondriacus, Vaccinium macrocarpon* and in *Tarenaya hassleriana* and, unexpectedly, also not in *Amborella trichopoda* and *Spirodela polyrhiza* (stippled lines). A more detailed analysis of Ericales (boxed) is given in Fig. 4. Also indicated is a parsimonious explanation for the presence of editing target ndhAeU473SL, which we discuss as an additional candidate target explaining retention of CLB19 in the Poales

Since we had observed an overall higher amount of chloroplast RNA editing but also a higher diversity of editing patterns in early-branching angiosperms as compared to model systems like *Arabidopsis thaliana,* we first investigated RNA editing also in *Illicium oligandrum* (Austrobaileyales) and *Chloranthus spicatus* (Chloranthales), representing other early-emerging flowering plant lineages. This clearly revealed that the *clpP* and *rpoA* mRNAs are typical examples for chloroplast genes more affected by RNA editing in the early-branching lineages with three or even four (in *Illicium*) additional sites of editing in *clpP* and *rpoA* (Fig. 2). Notably, the additional editing sites (clpPeU82HY, rpoAeU521SF and rpoAeU830SF) but not the CLB19 target edits clpPeU559HY and rpoAeU200SF are shared with gymnosperms, suggesting that the latter (and CLB19 as their cognate editing factor) originated early in the angiosperm stem lineage. Our expanded angiosperm sampling indeed revealed conservation of chloroplast RNA editing targets clpPeU559HY and rpoAeU200SF, and congruently of CLB19 orthologues, in the flowering plants with some notable exceptions (Fig. 3).

C-to-T conversions making editing clpPeU559HY obsolete are identified in *Vicia faba, Cicer arietinum, Carica papaya* and *Silene conica* (Fig. 3). Additionally, a phylogenetically deep loss of editing site clpPeU559HY has likely taken place in the “core” asterids (Cornales, Ericales, Solanales, Gentianales, Lamiales, Asterales and Apiales) after split from the Caryophyllales. Independent losses of editing site rpoAeU200SF have occurred in the plastomes of *Ziziphus jujuba*, *Eucalyptus grandis, Bienertia sinuspersici, Amaranthus tricolor* and *Silene latifolia*. Fully consistent with the findings of highly variable RNA editing in the mitochondrial transcriptomes in the genus *Silene* [19, 20], we also observe the most dramatic differences here for the two *Silene* species investigated. Whereas rpoAeU200SF is lost in *Silene latifolia* and clpPeU559HY remains to be edited, exactly the opposite is observed in *Silene conica*.

Unequivocal CLB19 orthologues are consistently identified in all the above taxa that have retained the one or the other of the two known CLB19 editing targets (Fig. 3, Additional file 3), hence fully congruent with the observations for CRR28 (Fig. 1). However, no CLB19 orthologues could be detected in the early branching taxa *Amborella* and in *Spirodela*. Remaining gaps in genome and transcriptome data can certainly not be fully excluded but given the overall high quality of available sequence data for these two species we consider this unlikely. CLB19 could indeed be obsolete in *Spirodela* as clpPeU559HY is “pre-edited” with a T being present in the cpDNA (Fig. 3) and rpoAeU200SF has been reported to be edited to ca. 7–8% only [24], possibly via spurious side-activity of other editing factors. Notably, *rpoA* editing was found slightly reduced in VAC1 mutants [25]. Our independent cDNA analyses (see Additional file 4) likewise revealed only very marginal editing of rpoAeU200SF in *Spirodela polyrhiza* at best. Nevertheless, the case of a missing CLB19 orthologue in *Amborella* remains puzzling. Congruent with its absence from the genomic data, we were unable to obtain a PCR product for CLB19 from *Amborella* DNA. However, we succeeded to obtain an unequivocal amplification product for a CLB19 orthologue in *Illicium oligandrum* representing another early-branching angiosperm, as expected for the required editing in *clpP* and *rpoA* (Fig. 2).

Both CLB19 editing targets at the same time are lost in at least six cases (Fig. 3): in the Poaceae/Poales except *Ananas*, in *Amaranthus hypochondriacus* (Caryophyllales), in *Camptotheca acuminata* (Nyssaceae, Cornales), in *Vaccinium macrocarpon* (cranberry, Ericales), in the Apocynaceae (Gentianales) and in *Tarenaya hassleriana* (Brassicales). In three of the above cases – *Vaccinium macrocarpon*, *Amaranthus hypochondriacus* and *Tarenaya hassleriana* – the simultaneous loss of both editing sites has obviously resulted in the loss of CLB19 orthologues from the nuclear genomes (Fig. 3), analogous to the case of CRR28 in chickpea (Fig. 1). The *A. hypochondriacus* case is particularly intriguing given that a CLB19 orthologue is still present in the sister species *A. tricolor* where editing site clpPeU559HY is retained. This suggests a recent and fast loss of clpPeU559HY and CLB19 in *A. hypochondriacus.* Similarly, the absence of CLB19 in *Vaccinium* vs. its presence in *Camellia* may suggest quick disintegration of the editing factor upon secondary loss of rpoAeU200SF as its second editing target. Given the ready availability of plant material in the species-rich Ericales we explored this issue more closely and sampled further taxa representing six families of the Ericales.

### The Ericales case: Loss and regain of editing targets and the final loss of CLB19

**Fig. 4:**
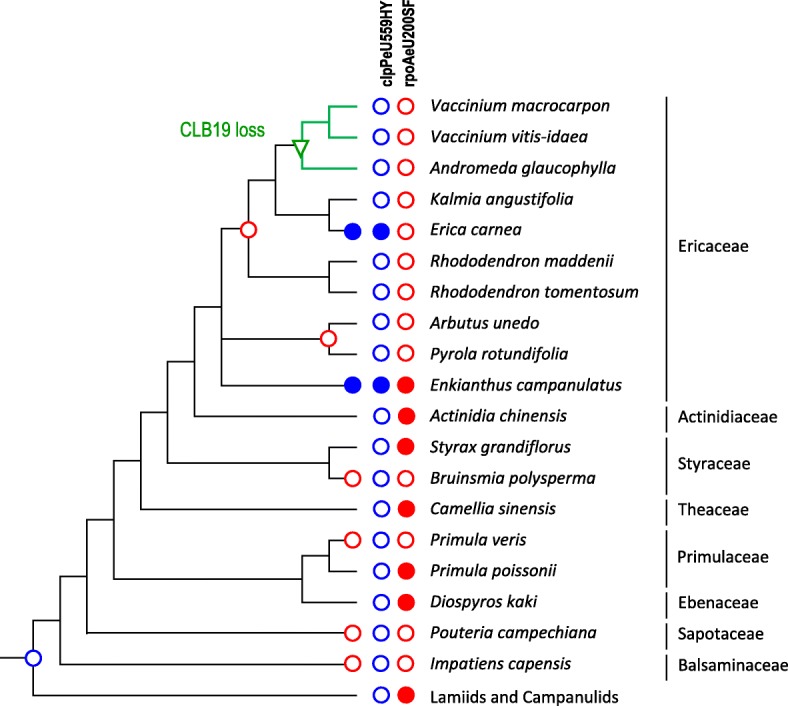
Cladogram of Ericales taxa (following most recent phylogenetic analyses [53]), for which chloroplast sequence information was obtained in this study (*Enkianthus, Rhododendron, Erica, Kalmia, Andromeda, Diospyros, Vaccinium vitis-idaea*) or was available in the NCBI database (*Impatiens, Pouteria, Bruinsmia, Styrax, Actinidia, Primula, Camellia, Pyrola, Arbutus, Vaccinium macrocarpon*). RNA editing site clpPeU559HY is ancestrally lost in the Asterid clade (blue open circle, see also Fig. 3). Editing site rpoAeU200SF is additionally lost in *Impatiens, Pouteria, Primula veris, Bruinsmia* and in the core Ericaceae (red open circles). Editing site clpPeU559HY is regained twice independently (blue filled circle). This ultimately inverts the ancestral editing status of Asterids in *Erica* and requires editing at both sites in *Enkianthus*. CLB19 is absent in *Andromeda* and *Vaccinium* (green triangle) as a likely secondary loss after loss of both its editing targets

The loss of the rpoAeU200SF editing site following the earlier loss of the clpPeU559HY editing target that was initially identified in *Vaccinium macrocarpon* (Fig. 3) appears to be a synapomorphy of the core Ericaceae (Fig. 4). An independent loss of rpoAeU200SF has occurred in *Impatiens capensis, Primula veris, Pouteria campechiana and Bruinsmia polysperma*. Surprisingly, we identified two cases (in *Enkianthus* and in *Erica*) where the ancestrally lost editing site clpPeU559HY has been regained (Fig. 4). Hence, RNA editing is now required at both sites in *Enkianthus* and for only the rpoAeU200SF site in *Erica*, thus inverting the ancestral state among early-branching Ericales or the Asterids at large. We used targeted PCR to specifically amplify CLB19 homologues in our Ericales DNA samples and were able to retrieve PCR products in *Enkianthus, Arbutus, Rhododendron, Erica* and *Kalmia* but not in *Andromeda* or in the two *Vaccinium* species. Evidently, these results suggest conservation of CLB19 if one target editing site remains (or is regained as in *Erica*) and the retention of the editing factor for a certain period of evolution after loss of both target sites (in *Rhododendron* and *Kalmia*) before its disintegration and loss (in *Andromeda* and *Vaccinium*).

### The case of CLB19 retention in the Poaceae

**Fig. 5:**
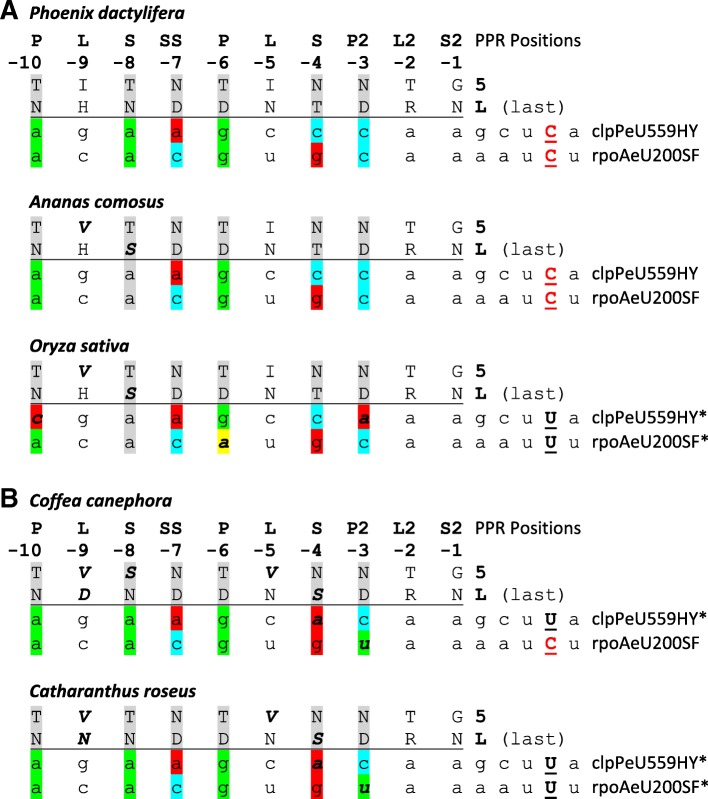
Alignment of editing site recognition sequences with crucial positions 5 and L (“last”) of the “PLS-type” PPRs in CLB19. The respective PPR-type (P, L, S, SS, P2, L2, S2) is indicated on top with numbering running backward, starting with the terminal S2-type PPR, which is juxtaposed with position − 4 upstream of the editing site (red underlined). Asterisks indicate loss of editing sites through C-to-T conversions in the monocot *Oryza* (panel **a**) or in the dicots *Catharanthus* and *Coffea* (panel **b**), respectively. Grey shading highlights P-, S-, SS- and P2-type repeats assumed to contribute to RNA recognition. Green nucleotide shading indicates perfect matches of PPR positions 5 and L according to strict canonical rules (T/S + N: A, T + D: G, N + N/S: C, N + D: U), blue shading indicates pyrimidine transitions, yellow shading indicates purine transitions and red shading indicates transversion mismatches, respectively. Changes from the presumed ancestral character states in CLB19 and the target sequences conserved in *Arabidopsis* and *Phoenix* are highlighted in bold and italics

The retention of CLB19 in all ten Poaceae species in our survey after loss of both its known editing targets (Fig. 3) is surprising given that the clade is dated to approximately 50–60 mio. Years (www.timetree.org, [21]). Accordingly, we inspected the CLB19 orthologues more closely (Fig. 5, Additional file 5). Interestingly, the loss of editing sites clpPeU559HY and rpoAeU200SF in the Poaceae is accompanied more by changes in the RNA target sequences rather than in the crucial RNA-binding amino acid positions 5 and Last (L) of the PPR motifs in the CLB19 orthologues as exemplified by the case of *Oryza sativa* (Fig. 5a). An A-to-C transversion and a C-to-A transversion in the region upstream of clpPeU559HY juxtaposed with PPRs P-10 and P2–3, respectively, and a G-to-A transition upstream of rpoAeU200SF opposite of P-6 all worsen target recognition according to the current rules of PPR-RNA interaction (Fig. 5). In contrast, changes in the relevant PPR positions are only observed for position 5 in PPR L-9 not believed to contribute to RNA binding and in position L of PPR S-8, which changes the canonical TN combination for recognition of adenine into TS. Moreover, these changes on the protein side are shared with *Ananas* as the closest outgroup within the Poales, which features both editing sites (Fig. 3).

The situation is similar in the case of the Gentianales (Fig. 5b) although more changes occur on the protein side affecting CLB19 PPRs L-9, S-8, L-5 and S-4. Again, however, changes in the target sequences appear more relevant than those in CLB19 itself. Loss of editing at clpPeU559HY in *Coffea* and *Catharanthus* is accompanied by C-to-A conversion seven nucleotides upstream of the former editing sites opposite of PPR S-4. Conversely, C-to-U transition six nucleotides upstream of the rpoAeU200SF edit (corresponding to PPR P2–3) would improve target recognition, but this change is shared between *Coffea* retaining the editing site and *Catharanthus*, which has lost both CLB19 editing targets.

## Discussion

The widely extended inspection of flowering plant genome and transcriptome data reported here has, on the one hand, fully corroborated the previous insights on editing factors CRR28 and RARE1 [17]. The single-target editing factor RARE1 is independently lost at least 20 times during angiosperm evolution upon loss of its editing target. The ancestral accDeU794SL RNA editing event converts a serine UCG codon into a leucine UUG codon. In the Caryophyllales lacking this edit (and concomitantly also RARE1, see Fig. 1), a “pre-edited” synonymous CTG leucine codon is found instead of a TTG codon in the cpDNAs. This could reflect a synonymous transition in the 1st codon position after loss of editing. However, we find that the ancestral serine-to-leucine edit has evolved into a proline-to-leucine edit converting a CCG into a CUG codon in the Cactaceae, another family of the Caryophyllales, possibly suggesting a different order of evolutionary steps here (not shown).

In contrast to RARE1, the dual-target editing factor CRR28 is retained also in all the now identified additional cases of losing either the one or the other of its editing site targets (Fig. 1). The loss of CRR28 in chickpea *Cicer arietinum* hence remains the only evident example for loss of CRR28 upon serial loss of both its editing sites.

**Fig. 6:**
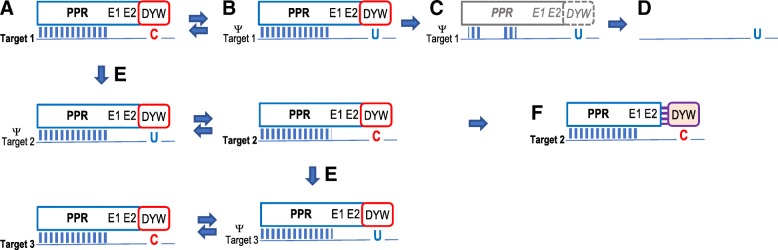
Scenarios for the evolution of DYW-type PPR protein editing factors. A single-target editing factor (**a**) may persist for some evolutionary time after cytidine-to-uridine conversion of its editing target (**b**) before functional disintegration (**c**) and ultimate loss (**d**). The numerous here reported independent losses of editing factor RARE1 among angiosperms (20 times) are examples for the latter case. The here reported retentions of RARE1 or CLB19 despite loss of their editing targets likely reflect state B rather than C given that no pseudogeniziation is recognizable. Editing factors may extend their functionality by acting on additional targets (**e**), likely allowing initial pseudo-targets to evolve into new editing sites by uridine-to-cytidine conversions. Once an editing factor serves multiple targets its loss depends on C-to-U conversion at all its targets simultaneously. The loss of CRR28 in *Cicer* or CLB19 in *Amaranthus, Tarenaya* and *Vaccinium* are examples. As an alternative to de-functionalization and loss, an editing factor may be functionally reduced to target recognition while the DYW domain is supplemented *in trans* (**f**). The here observed cases of CRR28 among Asterales are examples

Our expanded taxon sampling has now also unraveled cases for intermediate steps of evolution where an editing factor is kept for a certain period of evolutionary time after loss of its target, as to be expected (Fig. 6). RARE1 is retained without evidence for degeneration into a pseudogene in chestnut and oak despite C-to-T conversion at its original target site accDeU794SL and in *Trifolium subterraneum* despite *accD* gene transfer to the nucleus, likewise making editing obsolete (Fig. 1). The conservation of the key PPR residues (5 and L) in the RARE1 orthologues (not shown) provides no evidence for a target reassignment. Particularly in the light of a retained accDeU794SL editing in sister taxa, this issue will become more interesting with further genome data from the species-rich Fagales and Fabales.

**Fig. 7:**
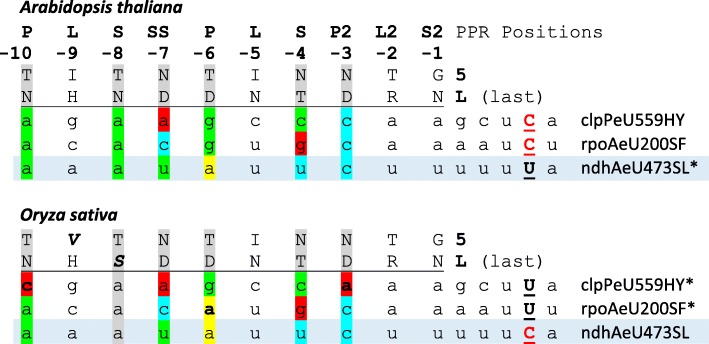
The ndhAeU473SL editing event is suggested as a candidate target of CLB19 orthologues among the Poaceae. Target nucleotides are shaded following the PPR-RNA recognition rules as in Fig. 5. Editing sites clpPeU559HY and rpoAeU200SF characterized as CLB19 targets in *Arabidopsis* are obsolete in rice and, vice versa, editing site ndhAeU473SL existing in rice as an additional candidate target for CLB19 is obsolete in *Arabidopsis* and all other eudicots

Here, we take the opportunity to suggest a designation for PPRs indicating their type – currently distinguished are P-, L-, S-, SS-, P2-, L2- and S2-type PPRs [5] – and numbering them backward starting from the canonical terminal S2-type PPR with “-1” (Figs. 5 and 7). The backward numbering has the advantage of putting more emphasis on the downstream PPRs that appear to contribute more significantly to RNA target recognition and avoiding number changes in occasional cases when revised protein models identify more upstream PPRs owing to loosened conservation or overlooked splicing. The additional annotation of amino acid identities for the key residues ‘5’ and ‘L’ then immediately allows to make prediction for the ribonucleotides that are expected to be targeted by a given PPR according to the canonical rule set [26], e.g. P-9TN, S-7NN, P-6TD or S-4ND likely targeting A, C, G or U, respectively.

It seems reasonable to assume that a complete DYW domain at the end of a PLS-type PPR protein represents an evolutionary ancient state. The assignment of editing sites to their respective editing factors is meantime complete in the moss *Physcomitrella patens* and all of its editing factors are canonical PLS-type PPR proteins with terminal DYW domains featuring the crucial cytidine deaminase signature residues at their end [14, 16, 27, 28]. Although ultimate biochemical proof is still lacking, the circumstantial evidence for the DYW domain being the cytidine deaminase is overwhelming [8–11, 29]. Evidently, editing factors that have lost the DYW domain *in cis* now rely on its supplementation *in trans* [30–33]. The now identified cases of CRR28 orthologues in the Asterales *Cynara* and *Lactuca* featuring degenerated DYW domains truncated at the PG box (Fig. 3) are likely examples for editing factors becoming functionally restricted to RNA target recognition (Fig. 6). Here, we can expect that a cytidine deaminase activity has to be provided *in trans*, either by direct protein-protein interaction or possibly mediated by MORF/RIP proteins [34, 35]. Interestingly, CRR28 had already been demonstrated to retain editing functionality after artificial deletion of its DYW domain behind the PG box [36], a scenario for which the Asterales now feature as a counterpart in natural evolution.

In contrast to editing factors CRR28 and RARE1 possessing a terminal DYW domain in nearly all angiosperms, CLB19 is an “E+”-type PLS protein lacking most of the DYW domain. Most recent studies confirmed that CLB19 editing relies on additional co-factors such as DYW2 and the extra PPR protein NUWA [32]. CLB19 was discovered early as the editing factor targeting chloroplast RNA editing sites clpPeU559HY and rpoAeU200SF [18]. A study of CLB19 targets among 21 Brassicaceae species revealed no losses of rpoA200SF or clpPeU559HY editing, but found that the rpoAeU200SF site may be edited as low as 40% in the steady-state chloroplast transcriptome [37]. Here we showed that CLB19 homologues are highly conserved in occurrence and structure in all angiosperms as long as one of its RNA editing targets remains present, very much like the CRR28 case. Only in the case of *Amborella trichopoda* we were unable to detect an expected orthologue, although both RNA editing sites were previously found to be efficiently edited [17]. Likewise, no CLB19 homologue was discovered in *Spirodela,* but it remains unclear whether this is due to insufficient genomic sequence quality or because the factor could be obsolete here as clpPeU559HY is pre-edited and rpoAeU200SF reported to be edited only to 7% or 8% in *Spirodela* [24].

Very much like in the CRR28 case, we identified several flowering plants retaining CLB19 if only the one or the other of its editing target site was lost (Fig. 3). Similarly, we found that CLB19 got lost once both its targets got lost owing to C-to-T transitions in the cpDNAs. However, in contrast to the only case of CRR28 loss in chickpea in the extended angiosperm sampling (Fig. 1), we found several cases for loss of CLB19 (Fig. 3). Among those, the case of *Amaranthus hypochondriacus* is intriguing given the retention of CLB19 and one of its targets (clpPeU559HY) in the sister species *A. tricolor* suggesting a rapid loss of CLB19 within the genus *Amaranthus.* Likewise, evolution of CLB19 and its targets is particularly interesting among the Ericales (Fig. 4). Not only does it reflect the serial losses of clpPeU559HY, rpoAeU200SF and finally of CLB19 (in *Vaccinium* and *Andromeda*) but also the retention of CLB19 after loss of both targets (in *Rhododendron* and *Kalmia*), which is to be expected as an evolutionary intermediate state (Fig. 6). Moreover, the Ericaceae also show two independent regains of the ancestrally lost clpPeU559HY edit, both before and after the later loss of edit rpoAeU200SF (Fig. 4). This results in the full spectrum of possible evolutionary states among Ericales with most taxa featuring CLB19 and edit rpoAeU200SF alone, at least one taxon featuring both edits (*Enkianthus*), at least one taxon featuring only edit clpPeU559HY (*Erica*), several genera retaining CLB19 without both targets (*Rhododendron* and *Kalmia*) and finally those having lost CLB19 after loss of the two targets (*Andromeda* and *Vaccinium*).

Whereas the above scenarios fit evolutionary expectations, the long-term retention of CLB19 despite an early loss of both known editing targets among the Poales likely calls for additional explanations. Although RNA-binding to its likely targets could initially not be demonstrated for CLB19 [18], it has later been selected for differential RNA-binding studies employing electrophoretic mobility shift assays (EMSAs) [38], also including successful alterations in its PPR array for retargeting [39]. Whereas Ramos-Vega and colleagues found comparable binding of CLB19 to both of its targets [38], the native *rpoA* target was found to be preferred over the *clpP* target in the EMSA studies by Kindgren et al. [39], somewhat unexpected from the prediction from the PPR-RNA binding code [26, 40, 41]. Changing the adenines juxtaposed with P-10TN and S-8TN into cytidines abolished binding to the *rpoA* target completely [38], as predicted (Fig. 5). A dinucleotide exchange upstream of the PPR recognition region on the other hand did not affect binding. However, another dinucleotide exchange including the conversion of the guanosine opposite of P-6TD into cytidine (Fig. 5), did very unexpectedly not affect binding either [38].

In contrast, on the protein side, PPR S-8TN of CLB19 showed low contribution in functional tests and this motif is found to be mutated for example to S-8TS in rice or to S-8SN in *Coffea* (Fig. 5). PPR SS-7ND proved to be much more important in the binding study. It showed the expected pyrimidine preference and is indeed highly conserved in our angiosperm sampling. On the other hand, although PPR P-10TN of CLB19 showed low contribution in the functional tests [39], it is now found to be highly conserved during flowering plant evolution, fitting the observations of the target mutation study [38].

Some further observations are noteworthy after a detailed compilation of the CLB19 PPRs and the respective targets for key taxa in our sampling (Additional file 5). While the matching adenines opposite S-8TN are widely conserved, this position is exceptionally mutated to C upstream of the lost editing target rpoAeU200SF in *Eucalyptus*. Likewise, cytidines conserved in *clpP* opposite of P2–3ND are mutated to non-matching adenines not only in Poaceae but also in *Sesamum indicum* and *Silene conica*, also having lost the clpPeU559HY editing target. Moreover, *Silene conica* features a guanosine upstream of the former clpPeU559HY edit, which is very rarely found in position − 1 immediately upstream of functional editing sites [42]. All of these alterations perfectly match the results for inhibited CLB19 binding to mutated targets [38]. The observations for position − 1 suggests that even an editing factor lacking a DYW-domain like CLB19 interacts with its RNA target at the 3′-end beyond its PPR array. The surprising conservation of the S2–1, L2–2 and L-5 PPR motifs (but not the L-9 PPR) in the CLB19 compilation (Additional file 5) furthermore suggests that the carboxyterminal PPRs, likely including the as yet enigmatic L-type PPRs, contribute to interaction with the 3′-end of target RNAs in ways that are not yet understood.

It has been noted early that a putative CLB19 ortholog exists in rice although the two targets in *Arabidopsis* would need no editing in the monocot owing to genomic C-to-T conversions [43]. Introducing the “aPPRove” program to predict PPR-RNA interaction, Harrison and colleagues suggested that CLB19 could also target a site in the second intron of the chloroplast *ycf3* gene previously identified as lowly edited in an *Arabidopsis* transcriptome study [44]. This hypothesis could easily be tested in the corresponding CLB19 mutant.

We here suggest an explanation for the retention of the CLB19 orthologue in Poales where both editing targets identified in *Arabidopsis*, rpoA200SF or clpPeU559HY, are lost owing to C-to-T conversions. Employing a new module (“TargetScan”) implemented in a new version of our PREPACT service (www.prepact.de) [42], we find that the ndhAeU473SL editing site documented in rice could be an alternative target of CLB19 (Fig. 7). Edit ndhAeU473SL is an ancient editing event, also shared with *Amborella trichopoda* [17], but lost early in the eudicot lineage, accordingly allowing for the here observed losses of CLB19 (Figs. 3 and 4). Hence, it will be highly interesting to investigate a KO line of the CLB19 orthologue in rice with respect to ndhAeU473SL editing in the future.

The cases of CLB19 retention among Apocynaceae in the Gentianales and in the *Camptotheca* lineage (Fig. 3) at present call for denser taxon sampling and forthcoming evaluation of the chloroplast editomes to decide whether they represent evolutionary intermediates or neo-functionalization of CLB19. In contrast to the Poaceae case, the retention of CLB19 among Ericaceae (Fig. 4) likely reflects the evolutionary intermediate stage towards disintegration and loss (Fig. 6). The here identified cases of regaining ancestrally lost editing sites among the Ericales (Fig. 4) reveal more complex and intricate pathways of PPR protein evolution among angiosperms than previously seen for RARE1 or CRR28.

## Conclusions

Extending an earlier sampling of angiosperms to investigate the co-evolution of chloroplast RNA editing and its nuclear-encoded specificity factors strongly supports previous insights but also identifies expected evolutionary intermediates (Fig. 6). Retention of an editing factor for some time after loss of its targets is now evident in at least two cases for single-target editing factor RARE1 and for three cases for the dual-target editing factor CLB19. Nevertheless, a similar scenario is not yet identified for CRR28, another dual-target chloroplast editing factor. CRR28 evolution among angiosperms, however, now reveals the loss of its terminal DYW domain as another evolutionary pathway. Retention of CLB19 after a deep loss of both its known editing targets in the Poaceae suggests additional functionality, possibly the monocot-specific editing event ndhAeU473SL. It will be interesting to see whether other dual-targeting editing factors with or without a terminal DYW domain will reveal similarly differing evolutionary scenarios like CRR28 and CLB19, respectively.

## Methods

### Collecting editing factor orthologues and phylogenetic analyses

*Arabidopsis thaliana* editing factors RARE1 (NP_196831) [43], CRR28 (NP_176180.1) [36] and CLB19 (NP_172066.3) [18] were used as protein queries in BLASTP and TBLASTN searches [45] against the angiosperm (magnoliophyte) data of the NCBI protein database, the TSA (Transcribed Shotgun Assemblies) and WGS (Whole Genome Shotgun sequences) databases, respectively (http://blast.ncbi.nlm.nih.gov/Blast.cgi). Qualities of genome and/or transcriptome data were evaluated for the presence of three conserved nuclear protein genes: PPR proteins VAC1/ECB2 and PDM1/SEL1 and the arginine decarboxylase ADC previously used for phylogenetic studies [46]. The quality screening ultimately resulted in a collection of 117 angiosperms (see Figs. 1 and 3 and main text) extending the previous sampling of 65 flowering plant species [17]. The MEGA alignment explorer [47] was used for sequence alignment and processing. Where necessary, nucleotide sequences were checked for possible sequence errors, manually translated and aligned with the other protein data. Gaps and missing or inaccurate C- and N- terminal sequences in erroneous protein models could frequently be improved. Special care was taken to avoid including editing factor paralogues. To this end, phylogenetic trees were re-checked for consistency with species phylogeny (see Additional files 1, 2 and 3) and individual sequences were checked to identify the respective *Arabidopsis* proteins as most similar homologues. Alignments initially obtained with the MUSCLE tool integrated in MEGA were manually edited. Alignments are available from authors upon request.

### Phylogenetic tree construction

Final alignments were used for calculation of ML (Maximum Likelihood) phylogenetic trees using the IQ-tree webserver (http://iqtree.cibiv.univie.ac.at/) [48]. The JTT + F + I + G4 model of sequence evolution was chosen for the CRR28 and RARE1 data sets and the JTT + I + G4 model for the CLB19 data set as the best-fitting models, respectively. Node reliability was determined from 1000 bootstrap replicates in each case.

### Collection of chloroplast sequences and RNA editing predictions

Wherever available, data from complete chloroplast genome assemblies were used. Nucleotide coding sequences for chloroplast target genes (*accD, clpP, ndhB, ndhD,* and *rpoA*) were ideally collected corresponding to the nuclear genome taxon sampling or taxonomically as closely as possible (marked by asterisks in Figs. 1 and 3) or newly determined during this study as outlined in the main text (e.g. for the gymnosperm outgroups or a denser Ericales sampling, see Figs. 2 and 4 and Additional file 4). In some cases, sequences were retrieved from WGS data (*Ensete, Eichhornia, Dianthus, Arachis, Quercus*, *Aquilaria, Linum, Citrus clementina* and *Metrosideros*) or data from available closely related sister taxa were employed as e.g. *Agave americana* or *Dianthus longicalyx*.

### Plant material and molecular work

Plant material for *Amborella trichopoda, Illicium oligandrum, Chloranthus spicatus, Ginkgo biloba, Cionura erecta, Vaccinium vitis-idaea, Andromeda glaucophylla, Kalmia angustifolia, Erica carnea, Rhododendron maddenii, Rhododendron tomentosum* and *Enkianthus campanulatus* was obtained from the Bonn University Botanic Garden. *Cycas taitungensis* was kindly provided by Christian Schulz from the Botanic Garden Bochum. *Diospyros kaki* and *Actinidia chinensis* were obtained from a local grocery store. Total plant nucleic acids were isolated using CTAB-based protocols [49, 50]. RNA preparations were alternatively obtained with the TRI reagent protocol (Sigma Aldrich). cDNA was synthesized with random hexamer or with gene-specific primers via the Revert Aid First Strand cDNA Synthesis Kit (Thermo Scientific/Fermentas). PCR amplicons for chloroplast genes or the *clb19* gene region were obtained using gene-specific primers (sequences available from the authors upon request) and Go-Taq polymerase (Promega) or Q5 polymerase (New England Biolabs) approaches. PCR products were isolated from agarose gels using the NucleoSpin Extract II Kit (Macherey & Nagel) and sequenced directly or after ligation into the pGEM-T Easy vector (Promega). Commercial Sanger sequencing was done by Macrogen Europe (Amsterdam, NL). A compilation of cDNA results obtained in the course of this work, in previous studies [17, 37, 51] or summarized in previous compilations [42, 52] is given in Additional file 4.

## Additional files

Additional file 1: Phylogeny of the CRR28 orthologs in angiosperms. Shown is a Maximum Likelihood tree (see Methods). (PDF 58 kb)

Additional file 2: Phylogeny of the RARE1 orthologs in angiosperms. Shown is a Maximum Likelihood tree (see Methods). (PDF 468 kb)

Additional file 3: Phylogeny of the CLB19 orthologs in angiosperms. Shown is a Maximum Likelihood tree (see Methods). (PDF 78 kb)

Additional file 4: Summary table on cDNA analysis for the RNA editing sites in question. (PDF 71 kb)

Additional file 5: Table of essential positions for RNA recognition in the PPRs of CLB19 and the corresponding RNA targets. (XLSX 40 kb)

## Abbreviations

EMSA: Electrophoretic Mobility Shift Assay
NCBI: National Center for Biotechnology Information
PCR: Polymerase Chain Reaction
PPR: Pentatricopeptide Repeat
TSA: Transcribed Shotgun Assemblies
WGS: Whole Genome Shotgun
