# Supplementary figures and images for: Expected and unexpected evolution of plant RNA editing factors CLB19, CRR28 and RARE1: retention of CLB19 despite a phylogenetically deep loss of its two known editing targets in Poaceae
Source: BMC Evol Biol. 2018 Jun 7;18:85. doi: 10.1186/s12862-018-1203-4 (PMC5992886; doi:10.1186/s12862-018-1203-4)

Additional File 1

CRR28 phylogeny

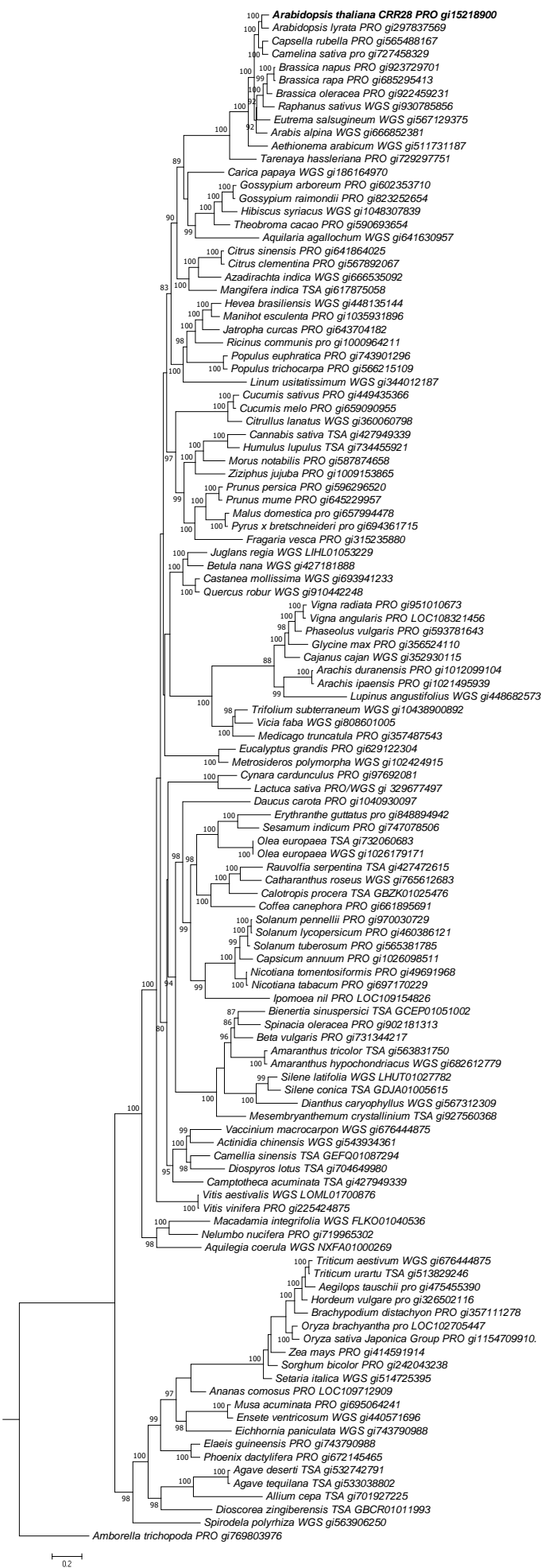

Supplement: Supplementary file 1 — Phylogeny of the CRR28 orthologs in angiosperms. Shown is a Maximum Likelihood tree (see Methods). (PDF 58 kb) [file 12862_2018_1203_MOESM1_ESM.pdf]

## Additional File 2

### RARE1 phylogeny

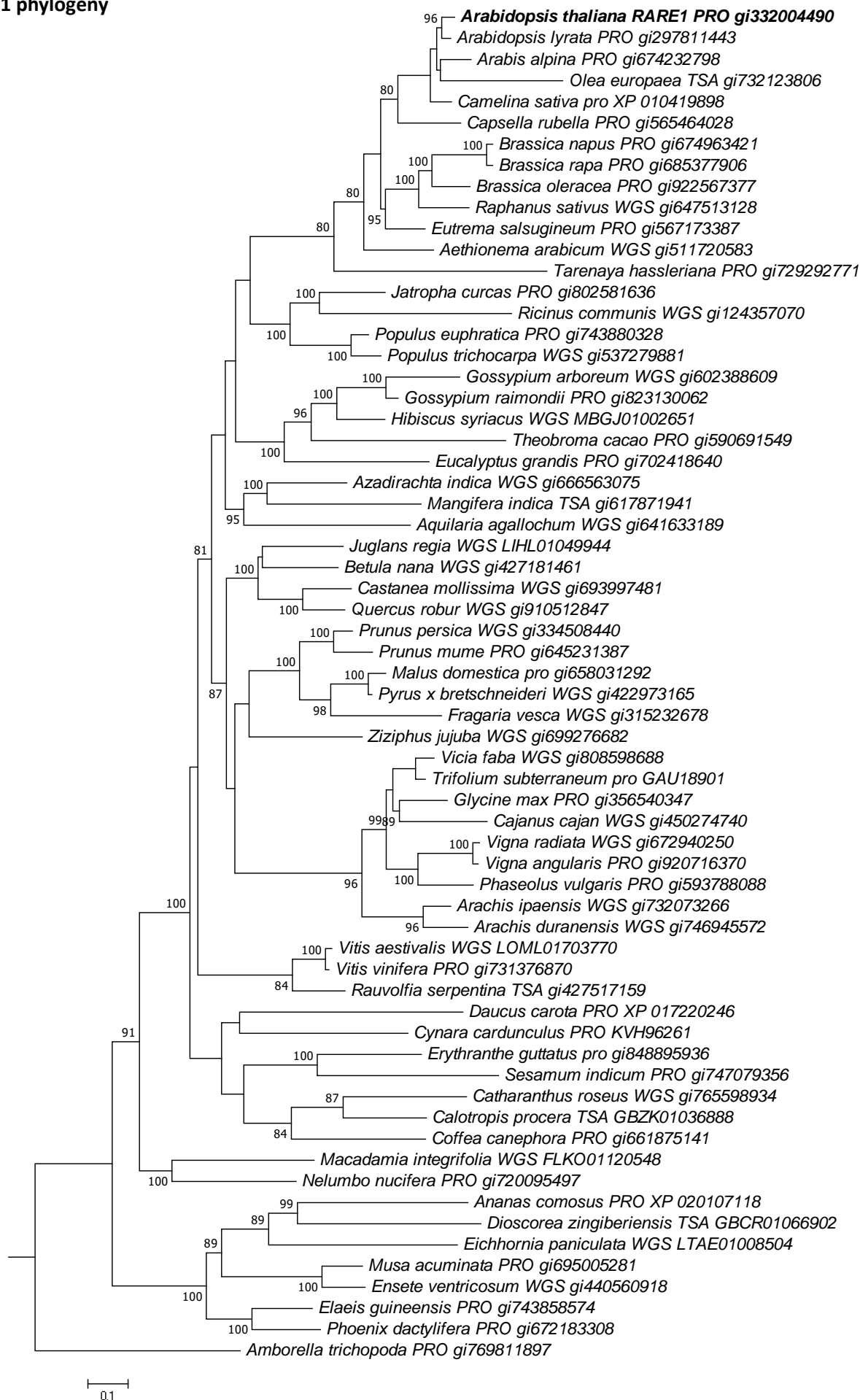

Supplement: Supplementary file 2 — Phylogeny of the RARE1 orthologs in angiosperms. Shown is a Maximum Likelihood tree (see Methods). (PDF 468 kb) [file 12862_2018_1203_MOESM2_ESM.pdf]

Additional File 3

CLB19 phylogeny

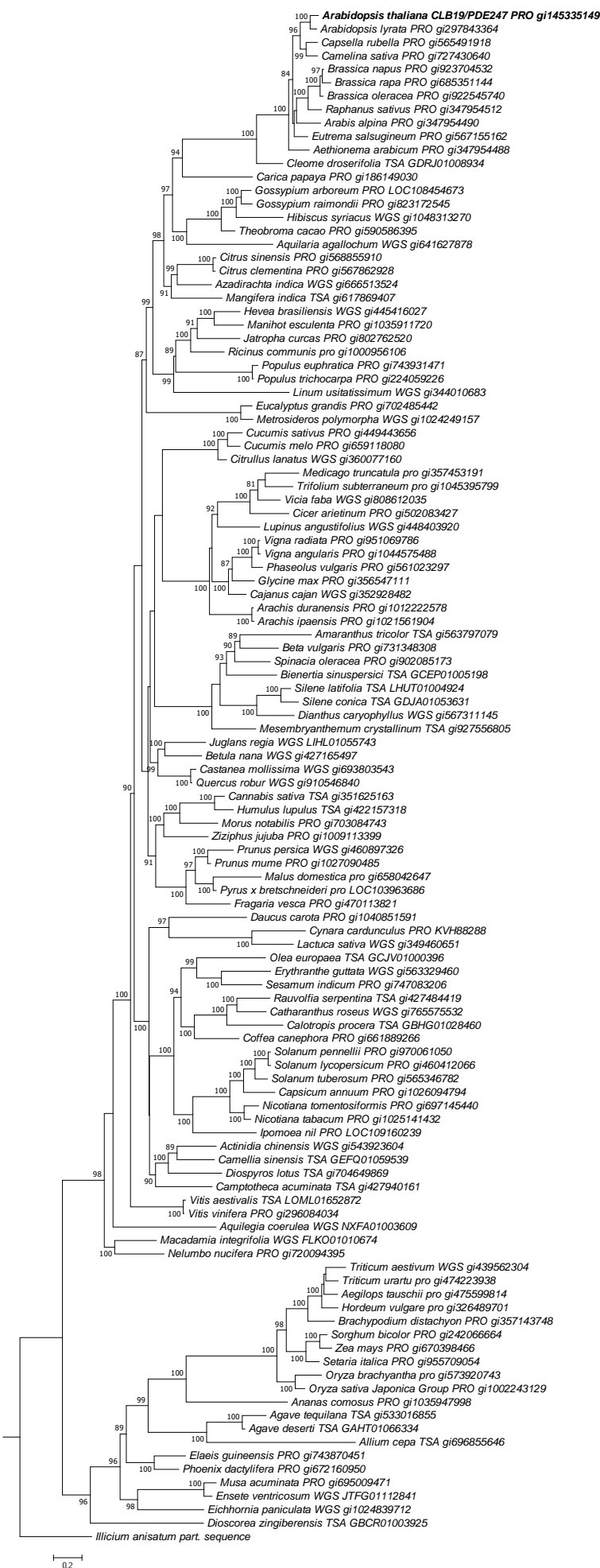

Supplement: Supplementary file 3 — Phylogeny of the CLB19 orthologs in angiosperms. Shown is a Maximum Likelihood tree (see Methods). (PDF 78 kb) [file 12862_2018_1203_MOESM3_ESM.pdf]
