# Supplementary material for: Expected and unexpected evolution of plant RNA editing factors CLB19, CRR28 and RARE1: retention of CLB19 despite a phylogenetically deep loss of its two known editing targets in Poaceae
Source: BMC Evol Biol. 2018 Jun 7;18:85. doi: 10.1186/s12862-018-1203-4 (PMC5992886; doi:10.1186/s12862-018-1203-4)
Supplement: Supplementary file 4 — Summary table on cDNA analysis for the RNA editing sites in question. (PDF 71 kb) [file 12862_2018_1203_MOESM4_ESM.pdf]

	accDeU794SL	ndhBeU467PL	ndhDeU878SL	clpPeU559HY	rpoAeU200SF	ndhAeU476SL	References
Arabidopsis thaliana	ed	ed	ed	ed	ed	p	[42]
Arabidopsis lyrata	ed	C	ed	ed	C	p	[52]
Capsella bursa-pastoris	C	ed	C	ed	ed	p	[37], this work
Eutrema salsugineum	C	C	ed	C	C	p	this work
Brassica rapa/oleracea	C	C	C	ed	ed	p	[37]
Raphanus sativus	C	C	C	ed	ed	p	[37]
Arabis hirsuta	C	C	C	ed	ed	p	[37]
Aethionema	C	C	C	ed	ed	p	[37]
Citrus sinensis	p	ed	ed	ed	ed	p	this work
Gossypium hirsutum	ed	ed	ed	ed	C	p	[42]
Hibiscus syriacus	C	C	C	C	ed	p	OneKP data
Cucumis sativus	p	ed	ed	ed	ed	p	[17, 42], this work
Betula nana	ed	ed	ed	ed	ed	p	this work
Vigna radiata	ed	p	ed	ed	ed	p	[42]
Phaseolus vulgaris	C	p	ed	C	C	p	[52], this work
Vigna angularis	C	p	ed	C	C	p	this work
Glycine max	ed	p	ed	ed	ed	p	[51], this work
Cajanus cajan	C	p	ed	C	C	p	this work
Vicia faba	C	p	ed	p	C	p	this work
Arachis	C	ed	ed	C	C	p	this work
Lupinus angustifolius	p	ed	ed	ed	ed	p	this work
Hevea brasiliensis	p	ed	ed	ed	ed	p	[42]
Vitis vinifera	C	ed	C	C	C	p	this work
Solanum lycopersicum	p	ed	ed	p	ed	p	this work
Nicotiana tabacum	p	ed	p	p	ed	p	[42],[52]
Daucus carota	C	ed	C	p	C	p	this work
Beta vulgaris	C	ed	ed	ed	ed	p	this work
Nelumbo nucifera	ed	ed	C	ed	ed	C	this work
Hordeum vulgare	-	ed	ed	p	p	ed	[52]
Oryza sativa	-	ed	ed	p	p	ed	[42]
Zea mays	-	ed	ed	p	p	ed	[42],[52]
Aegilops tauschii	-	ed	ed	p	p	ed	[42]
Ananas comosus	C	ed	C	ed	ed	p	this work
Allium cepa	p	ed	ed	C	ed	C	this work
Dioscorea zingiberiensis	C	ed	ed	ed	ed	C	this work
Spirodela polyrhiza	p	ed	ed	p	0-8%	ed	[42], this work
Illicium oligandrum	ed	ed	ed	ed	ed	ed	this work
Nuphar advena	p	C	ed	C	ed	C	this work
Calycanthus floridus	C	C	ed	C	ed	C	this work
Chloranthus spicatus	ed	ed	ed	ed	p	C	this work
Liriodendron tulipifera		C	ed	C	C	C	this work
Magnolia kwangsiensis	C	ed	ed	ed	ed	C	this work
Amborella trichopoda	ed	ed	ed	ed	ed	ed	[17]

Legend: **rence numbering as in manuscript:**

editing confirmed	ed	[42]: et al 2018
C in DNA, ed predicted	C	[17]ı et al 2016
editing not confirmed	C	[52]lice et al 2018
"pre-edited", T in DNA	p	[37]s et al 2012
		[51]ıes et al 2017
